# Altered platelet indices as potential markers of severe and complicated malaria caused by *Plasmodium vivax*: a cross-sectional descriptive study

**DOI:** 10.1186/1475-2875-12-462

**Published:** 2013-12-27

**Authors:** Fábio A Leal-Santos, Soraya BR Silva, Natasha P Crepaldi, Andréia F Nery, Thamires OG Martin, Eduardo R Alves-Junior, Cor JF Fontes

**Affiliations:** 1 Malaria Clinic, University Hospital of Federal University of Mato Grosso, Rua Luis Phelippe Pereira Leite, s/n, Alvorada, Cuiabá (MT) CEP: 78048-902, Brazil

**Keywords:** Malaria, *Plasmodium vivax*, Platelet indices

## Abstract

**Background:**

This study described altered platelet indices in patients with acute malaria caused by *Plasmodium vivax* and determined whether these alterations are associated with warning signs of severe and complicated malaria.

**Methods:**

A total of 186 patients attending the Malaria Clinic at the University Hospital from the Federal University of Mato Grosso, Brazil, between 2008 and 2013 were included in this study. After parasitological confirmation of exclusive infection by *P. vivax*, blood cell counts and platelet indices were determined. Disease gravity was evaluated on the basis of classic signs of *Plasmodium falciparum* severe malaria, including severe anemia, or by changes in serum levels of glucose, bilirubin, aminotransferases and creatinine at the time of the patient’s admission. Patients with a longer duration of symptoms or those identified as primo infected were considered potential candidates for evolution into the severe form of malaria.

**Results:**

The mean platelet volume (MPV), platelet distribution width (PDW), and plateletcrit (PCT) values exhibited significant variability. A significant inverse relationship was observed between parasitaemia and PCT. Patients with warning signs for evolution into severe disease, with primo infection, or presenting with symptoms for over three days had the highest MPV and PDW. The adjusted analyses showed the presence of warning signs for the development of severe and complicated malaria remained independently linked to elevated MPV and PDW.

**Conclusion:**

Altered platelet indices should be analysed as potential markers for the severity of malaria caused by *P. vivax*. Future studies with appropriate methodology for prognostic evaluation could confirm the potential use of these indices in clinical practice.

## Background

Several clinical complications have been described in malaria caused by *Plasmodium vivax*, including severe anaemia, cerebral malaria, acute pulmonary oedema, and multi-organ failure [1]. Although thrombocytopaenia is often reported, the occurrence of bleeding is rare in these patients [2].

Changes in platelet counts during acute malaria are commonly reported in the medical literature, especially in *Plasmodium falciparum* infections; such changes are a major cause of concern to clinicians because such cases are more likely to evolve into serious and complicated disease cases [3,4]. However, many recent studies have also found thrombocytopaenia associated with *P. vivax*[5-7]. In general, the underlying mechanisms of thrombocytopaenia in malaria are peripheral destruction, excessive sequestration of platelets in spleen, and excessive use of platelets associated with the disseminated intravascular coagulation phenomenon [8]. In addition to the reduction in the number of platelets, platelet function is also compromised in these patients; this is generally evidenced by changes in the volume and other features of platelet cells [9].

Moreover, in addition to their function in haemostasis, platelets play an important role in the inflammatory response [10]. Changes in platelet counts during bacterial infections are reported to be associated with enhanced disease severity and mortality [11]. Furthermore, platelet activation alters the morphology of these cells, which can be evaluated on the basis of mean platelet volume (MPV) and platelet distribution width (PDW) [12]. Another platelet parameter is plateletcrit (PCT), which is a reliable measurement of platelet biomass because it combines the MPV with the absolute platelet count [13]. MPV, PDW and PCT can be altered in patients with cardiovascular [13] and infectious diseases such as pulmonary tuberculosis [14]. All of these indices are considered markers of platelet activation [9,15] and are altered in different clinical conditions [16-18]. Nevertheless, little is known about the alterations in these indices in malaria, especially in infections with *P. vivax*.

Some studies reported MPV to be elevated in malaria [4,19,20]. Furthermore, the validity of this finding for the diagnosis of acute malaria was recently tested in suspected cases in India [21]. However, the relationship between this increase and the clinical outcome of malaria infection remains controversial [19,22]. Furthermore, there is no available information about the relationship between altered PDW or PCT and the severity of malaria caused by *P. vivax*.

The present study aimed to determine the frequency and factors associated with alterations in platelet indices in patients with malaria caused by *P. vivax*, as well as their associations with clinical and laboratory indicators of severe and complicated malaria outcomes.

## Methods

This is a cross-sectional descriptive study based on the clinical and laboratory data of 186 patients with acute malaria caused by *P. vivax* who attended the Malaria Clinic at the University Hospital of the Federal University of Mato Grosso between 2008 and 2013. Data were collected on both ways: on a standardized form from 50 patients and extracted from the records of the remaining ones. Patients with exclusive *P. vivax* infection were eligible to participate in this study. All the patients underwent haemogram and blood biochemical analyses at their first appointment. Malaria was diagnosed on the basis of the microscopic examination of Giemsa-stained thick smears. All blood cell counts were determined using a same automated equipment (Pentra 80; Horiba Medical, Montpellier, France), which provides results regarding MPV, PDW, and PCT. The normal ranges for MPV, PDW and PCT provided for this equipment are 7.0/μm^3^-10.5/μm^3^, 11%-18% and 0.15%-0.50%, respectively.

Blood samples were continuously agitated after collection. To avoid interference in the analysed parameters, the time between blood sample collection and cell counting was ensured to be shorter than 60 minutes. All the participants signed an informed consent and underwent a detailed clinical evaluation by a team physician prior to participating. Information about the number of previous malaria infections and time of symptom onset was collected from all the patients.

Potential severe and complicated malaria caused by *P. vivax* was defined by the presence of one or more of the classic signs used as predictors of *P. falciparum* severity recommended by the World Health Organization [23] or the following changes in blood haematology or biochemical parameters at the time of patient’s admission: body temperature ≥41°C, dyspnoea, arterial hypotension, serum creatinine level >1.5 mg/dL, hypoglycaemia, hyperbilirubinaemia and haemoglobin <7 g/dL or haematocrit <20%. Moreover, primo infection and reporting symptoms that persisted for longer than three days were also considered potential risk factors for evolution into severe disease.

The data were analysed by the Stata version 12 software. The Mann-Whitney non-parametric test was used to analyse the association between platelet indices with the presence/absence of markers for severe or complicated malaria caused by *P. vivax*. Spearman’s correlation coefficients were calculated to evaluate the relationships between platelet indices and parasitaemia. Multivariate logistic regression models were constructed with each parameter as a dependent variable to analyse the independence of the associations between severity and platelet indices. For this purpose, MPV, PDW, and PCT were stratified into two groups, namely above and below the median. The reason to use these strata was the large range of the normal platelet indices. The backward stepwise method was used to include all the studied variables in the model. The level of significance was set at *p* <0.05. This study was approved by the Research Ethics Committee of Julio Müller University Hospital (no. 242,721).

## Results

The patients had a median age of 37,5 years and mean (SD) of 37.7 (14.7) years, and 145 (78.0%) were men. The elapsed time between symptom onset and the diagnosis of malaria ranged from one to 60 days (median = 5 days), with a mean (SD) of 7.1 (8.6) days. However, malaria was only diagnosed after 96 hours (i e, four days after symptom onset) in 54.4% of patients. At the time of diagnosis, 56.1% of the patients had fever. Twenty-one patients (11.3%) had at least one warning sign of severe and complicated malaria, including hyperpyrexia, severe anaemia, arterial hypotension, and elevated serum creatinine. The mean parasitaemia level observed in 186 patients was 7,256 (11,147) parasites per mm^3^ blood (median = 4,186). No patient reported using medicines with the potential to interfere with platelet indices (Table 1).

**Table 1:** **Clinical and laboratory characteristics of patients with malaria caused by *Plasmodium vivax***

**Characteristics**		***n***	**%**
Age (years) (*n* = 182)	*0-12*	5	2.7
	*13-49*	139	76.4
	*≥50*	38	20.9
		Mean (SD), 37.7 (14.7)	
Sex	*Male*	145	78.0
	*Female*	41	22.0
Days with symptoms (*n* = 167)	*1*	18	10.8
	*2-4*	58	34.7
	*>4*	91	54.5
		Mean (SD), 7.1 (8.6)	
Fever at diagnosis (*n* = 157)	*Yes*	88	56.1
	*No*	69	43.9
Indication of severity	*Yes*	21	11.3
	*No*	165	88.7
Parasitaemia/mm^3^ (*n* = 172)		Mean (SD), 7,256 (11,147)	

Variation in the numbers of individuals is due to missing information.

The haematological evaluation of all the patients showed a mean haemoglobin level of 12.9 (2.0) g/dL, mean haematocrit level of 38.5% (5.5%), and mean leukocyte count of 6,278 (6,323) leukocytes/mm^3^. The mean platelet count was 114,823 (76,761) cells/mm^3^; 16.7 and 60.7% of the patients exhibited counts fewer than 50,000/mm^3^ and between 50,000/mm^3^ and 150,000/mm^3^, respectively. The MPV was 9.3 (1.0) μm, mean PDW was 17.5% (3.36%), and mean PCT was 0.104% (0.066%). All the blood haematological parameters were done in a same automatic equipment.

**Figure 1:**
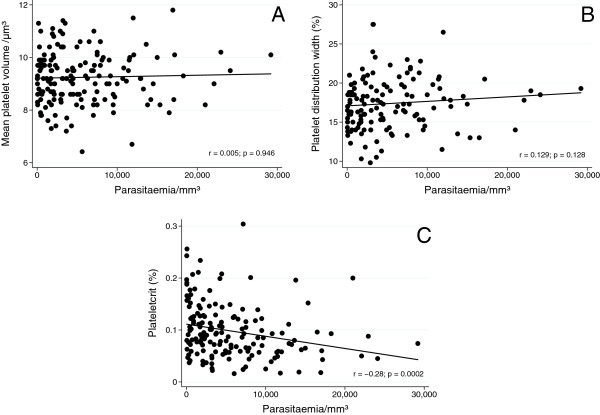
**Relationships between parasitic density and other variables in the patients with malaria caused by *Plasmodium vivax*.** Parasitic density *versus* MPV **(A)**, PDW **(B)**, and PCT **(C)**.

Potentially more serious cases, that is, primo-infected patients, exhibited significantly higher MPV and PDW (*p* = 0.0003 and *p* = 0.0003, respectively) and signs of gravity (*p* = 0.042 and *p* = 0.036, respectively). Conversely, patients with three or more days of symptoms had lower PCT (*p* = 0.045; Table 2). A weak but statistically significant negative correlation was observed between parasitaemia and PCT (r = -0.28; *p* = 0.0002). However, no significant correlation was observed between parasitaemia and MPV or PDW (Figure 1).

**Table 2:** **Associations between platelet indices and clinical signs of severity in the patients with *Plasmodium vivax *** **malaria**

		**Platelet parameter**		
		**Mean (SD)**		
		**MPV (per μm**^**3**^**)**	**PDW (%)**	**PCT (%)**
**First infection**	*Yes*	9.77 (0.98)	19.87 (4.61)	0.086 (0.046)
	*No*	9.09 (0.94)	16.85 (2.89)	0.102 (0.053)
	*p*	0.0003	0.0003	0.1165
**Time of symptom onset**	*>3 days*	9.32 (1.03)	17.93 (3.75)	0.091 (0.055)
	*≤3 days*	9.12 (0.86)	16.77 (2.81)	0.104 (0.044)
	*p*	0.166	0.065	0.045
**Warning signs for severity**	*Present*	9.62 (1.12)	18.98 (3.64)	0.079 (0.036)
	*Absent*	9.21 (0.95)	17.35 (3.46)	0.105 (0.067)
	*p*	0.042	0.036	0.084

*p* values were calculated according to the Mann-Whitney test.

Adjusted models were constructed for each platelet parameter, which were categorized as higher or lower the median. Variables exhibiting significant associations in the univariate analysis were included in the model in addition to age and sex. After adjustments, primo infection and the presence of warning signs for severe malaria were independently associated with the highest MPV. Conversely, PDW remained associated with the number of previous malaria infections and the presence of signs of gravity. Only the longest duration of symptoms remained associated with the lowest PCT (Table 3).

**Table 3:** Multivariate logistic regression analysis of the associations between platelet indices, demographic variables, and severity indicators

**Variable**	***Odds ratio***	***95% CI***	***p***
***MPV***			
Primo infection	2.72	1.16-6.38	0.039
Presence of warning signs for severity	3.47	1.02-11.75	0.033
***PDW***			
Previous episodes of malaria	0.91	0.85-0.97	0.004
Presence of warning signs for severity	5.44	1.20-24.78	0.028
***PCT***			
Time with symptoms (days)	2.46	1.11-5.46	0.038

Variables showing *p* ≤0.20 in the univariate analysis were included in the model in addition to age and sex.

## Discussion

In the present study, the analysis of the platelet indices in 186 patients with malaria caused by *P. vivax* revealed a high frequency of thrombocytopaenia and changes in MPV, PDW, and PCT. The high frequency of warning signs of severe malaria cases can be explained by the fact that this study was conducted in a reference hospital for malaria diagnosis and treatment.

MPV was higher than the median value in 46.2% of the patients, which is a higher percentage than that observed in patients infected with *P. falciparum* (25%) in Saudi Arabia [24] but similar to that reported by Franklin *et al.*[6] and Coelho *et al*. [20] regarding patients infected with *P. vivax* in the Amazon region. Increased MPV in malaria has been observed in other studies [4,19]. The validity of using such an increase in MPV in the diagnosis of acute malaria in suspected cases was recently tested in India; the authors concluded that a MPV exceeding 8 μm^3^ exhibits a sensitivity and specificity of 70.8 and 50.4% for the diagnosis of malaria, respectively [21].

It is well known that non-immune individuals are more susceptible to developing severe malaria. Furthermore, the delay of onset of malaria treatment is directly associated with severe disease outcomes [23]. In this study, changes in platelet indices predominated in the patients with any indicator of severe malaria caused by *P. vivax*, such as primo infection, longer symptom duration, and the presence of clinical signs and laboratory indicators of severe malaria. Larger platelets are metabolically and enzymatically more active and have a more important role in the inflammatory process [25]. Elevated MPV has also been described in patients with severe sepsis and is explained by the quick splenic and medullary release of large volumes of platelets in response to the increased demand for these cells [16]. In fact, studies in humans and rats showed that large platelets are functionally more active and have a lower threshold for aggregation and the release of their activity [9,16]. In bacterial diseases, increased MPV is associated with invasive infection or refractoriness to antimicrobial treatment [26].

It is well known that PDW is linearly correlated with MPV in normal individuals [12]. However, it is unlikely that the increased PDW observed in the present study was solely a consequence of increased MPV. Platelet width is reported to be greatly heterogeneous even with normal MPV in myeloproliferative diseases [12], eclampsia [27], acute coronary syndrome and heart failure [28], vascular micro-occlusion of sickle-cell disease [29], and bacteraemia [9,16,26]. Only one study mentions MPV in malaria and concluded that this parameter can be considered a predictor of acute infection by *P. falciparum*[21]. In all of these studies, the platelet activation was considered the main mechanism inducing the elevation of MPV and PDW.

Low mean PCT was also verified in the patients with acute malaria in the present study. However, little is known about PCT during the courses of various diseases. Thus, PCT is the most neglected haematological parameter in clinical practice and one of the least often reported in publications in the biomedical literature. PCT alterations are observed in some clinical conditions such as coronary ischemia [13], diabetes mellitus [30], pulmonary tuberculosis [31], sleep apnea [32], and inflammatory bowel disease [33].

The preference of *P. vivax* for infecting only young cells in peripheral blood avoids the occurrence of high parasitic density in vivax malaria [34]. However, we found a negative correlation between the level of parasitaemia and PCT. Once the PCT is a measure of the platelet biomass, this finding may be explained by the concomitant thrombocytopenia, which is also known to be associated with thrombocytopenia associated with *P. vivax* infections [35,36].

This study has some limitations that should be mentioned. First, other conditions causing platelet count reduction and changes in platelet indices were not systematically investigated. However, the changes in the studied parameters as part of post-treatment evaluation suggest that malaria was the most likely cause of the observed alterations.

## Conclusion

In conclusion, this study shows that platelet indices were altered during acute and symptomatic infection by *P. vivax*. The elevation of MPV and PDW, and reduction of PCT are related to known potential risk factors for evolution into severe malaria, such as primo infection, longer symptom duration, and the presence of the classical warning signs of severe and complicated *P. falciparum* malaria. Therefore, these parameters and indices could be useful as predictors of severity in the clinical approach of patients with malaria caused by *P. vivax.* Nevertheless, studies with appropriate methodology to measure prognostic outcomes are required to confirm the efficacy of these indices in clinical practice.

## Competing interests

The authors declare that they have no competing interests.

## Authors’ contributions

FALS coordinated specimen collection and analysis of clinical specimens, statistical analysis, manuscript writing and participated in study design. TOGM and ERAJ participated in specimen collection and oversight for malaria microscopy. AFN, SBRS and NPC performed the clinical evaluation of the included patients. CJFF conceived the study, participated in study design, data analysis, manuscript writing and was the lead study investigator. All authors read and approved the final manuscript.
